# Resection of Liver Metastases: A Treatment Provides a Long-Term Survival Benefit for Patients with Advanced Pancreatic Neuroendocrine Tumors:* A Systematic Review and Meta-Analysis*

**DOI:** 10.1155/2018/6273947

**Published:** 2018-11-14

**Authors:** Xinzhe Yu, Jichun Gu, Haoxuan Wu, Deliang Fu, Ji Li, Chen Jin

**Affiliations:** ^1^Department of Thoracic Surgery, Fudan University Shanghai Cancer Center. 270 Dong-An Rd, Shanghai 200032, China; ^2^Department of Oncology, Shanghai Medical College, Fudan University, Shanghai, China; ^3^Pancreatic Surgery Department, Huashan Hospital Affiliated to Fudan University, Shanghai 200040, China

## Abstract

**Purpose:**

Nonsurgical therapies, including biotherapy, chemotherapy, and liver-directed therapy, provided a limit survival benefit for PNET patients with hepatic metastases. With the development of liver resection technique, there was a controversy on whether to perform a liver resection for these patients.

**Methods:**

A computerized search was made of the Medline/PubMed, EMbase, Cochrane Library, and SinoMed (CBM) before March 2018. A meta-analysis was performed to investigate the differences in the efficacy of liver resection and nonliver resection treatments based on the evaluation of morbidity, 30-day mortality, symptom relief rate, and 1-, 3-, and 5-year survival. Two investigators reviewed all included articles and extracted the data of them. The meta-analysis was performed via Review Manager 5.3 software.

**Results:**

A total of 13 cohort studies with 1524 patients were included in this meta-analysis. Compared with the nonliver resection group, liver resection group had a longer 1-, 3-, and 5-year survival time and a higher symptom relief with an acceptable mortality and morbidity.

**Conclusions:**

Liver resection is a safe treatment and could significantly prolong the long-term prognosis for highly selected patients with resectable liver metastases from PNET. Further randomized, controlled trials are needed.

## 1. Introduction

PNET (pancreatic neuroendocrine tumor), commonly known as islet cell tumors, is a rare malignant neoplasm comprising of <2% pancreatic tumors and its incidence is <1 per 10,000 person per year [1–3]. However, the incidence is increasing recently due to the advancements of imaging and endoscopic technique [4]. In contrast to pancreatic adenocarcinoma, PNET is a kind of relatively indolent tumor [5]. PNET is highly heterogeneous and could be separated with many different subtypes according to secreted hormones [6]. Owing to 50%-80% of PNETs are malignant (except for insulinomas), metastases always turn out during the progression of PNETs and liver is a frequent disseminate site [5, 6] Treatments of hepatic metastases include surgery (hepatic resection), intervention (embolization [HAE] and transcatheter arterial chemoembolization [TACE]), biotherapy (octreotide/interferon and peptide receptor radionuclide therapy [PRRT]), systemic chemotherapy (streptozotocin, 5-fluorouracil, and everolimus), and ablation. Chemoembolization means HAE combined with chemotherapeutic agents [5, 7, 8]. Among these approaches, liver surgery for metastatic disease has provided a potentially curative choice for patients with colorectal cancer [9]. With the safety enhancement of liver surgical techniques, hepatic resection is becoming an optimal option for PNET patients with liver metastases [10]. This meta-analysis was mainly to evaluate overall survival outcomes and postoperative symptom relief from PNET patients with liver metastases between liver resection and nonliver resection groups.

## 2. Materials and Methods

### 2.1. Search Strategy

**Figure 1:**
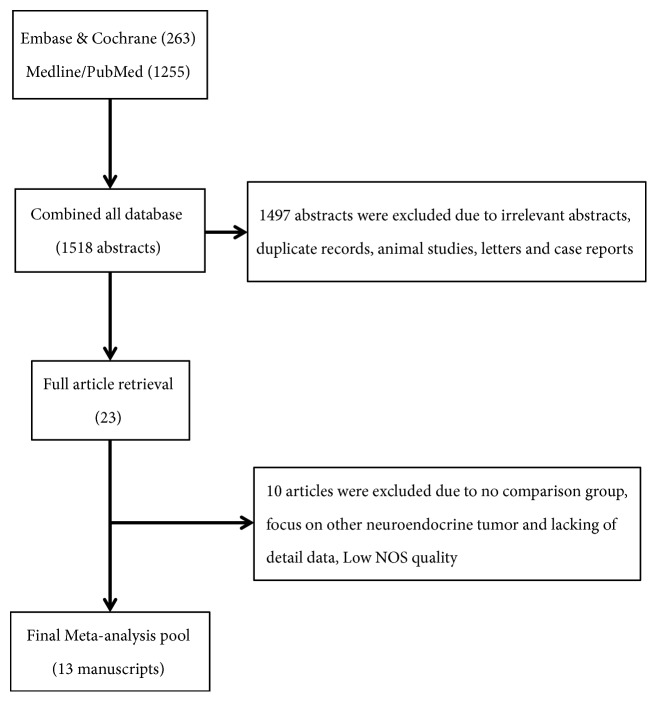
PRISMA Flowchart describing literature search history.

A computerized search was made of the Medline/PubMed, EMbase, Cochrane Library, and SinoMed (CBM) before March 2018. No language was limited. We used the following keywords: “pancreatic neuroendocrine tumors”, “hepatic metastases”, “liver metastases”, “hepatic metastases resection”, “liver metastases resection”, “hepatectomy”, “hepatic resection”, and “liver resection”, and we combined these keywords with “AND” “OR”. We also searched related references in the retrieved studies and reviewed articles from the database. For details, please see the flowchart of search history in Figure 1.

### 2.2. Inclusion and Exclusion Criteria

The original studies included in the meta-analysis need to meet the following criteria: (1) cohort or comparative studies of patients with liver metastatic PNET undergoing hepatectomy; (2) at least 10 patients that should be reported; (3) at least 1-year overall survival (OS) data that should be available after hepatic resection; (4) NOS score≥6. Abstracts, letters, animal experiments, reviews without original data, case reports, and studies lacking control groups were excluded.

### 2.3. Data Extraction

Abstracts of all articles were identified by two reviewers (Xinzhe Yu & Jichun Gu) independently. If there were any discrepancies which could not be solved with discussion between the two authors, a third independent author (Chen Jin) would determine the eligibility and data of the study. We extracted such data from all articles as follows: first author, year of publication, study population characteristics, study design, inclusion and exclusion criteria, resection margin, procedure-related morbidity and mortality, OS, and median follow-up. All of the texts, tables, and figures were reviewed for data extraction. All patients in liver resection group were treated with the resection of primary tumors and liver metastases. Patients in nonliver resection group underwent nonliver resection with or without primary tumors resection.

### 2.4. Quality Assessment

A quality assessment of retrieved studies in this meta-analysis was carried out in the form of Newcastle-Ottawa Quality Assessment Scale (NOS System) for cohort studies [11]. The aspects of selection, comparability, and follow-up were assessed with every inclusive study. Any study that could obtain a score ≥ 7 may be recognized as high-quality study for inclusion.

### 2.5. Statistical Analysis

We conducted the meta-analysis following the MOOSE guidelines [12] with the Review Manager 5.3 software. The outcomes of liver resection group versus nonliver resection group were pooled with a random-effect or fixed-effect meta-analysis. In addition, heterogeneity among studies was evaluated by I^2^ and *p* value, with significance being set at* p*< 0.05 and I^2^ > 50%. A high value for I^2^ indicates heterogeneity. If there was substantial heterogeneity, random effects models would be used in analysis. Publication bias was evaluated with a funnel plot.

## 3. Results

**Figure 2:**
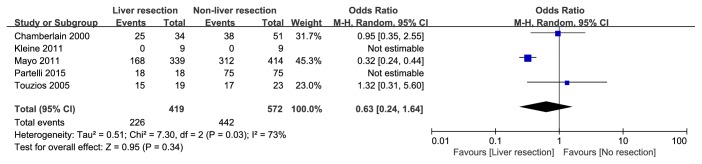
Forest plot for the occurrence time of hepatic metastases (synchronous/metachronous) of liver resection group and nonliver resection group in patients with liver metastases from pancreatic neuroendocrine tumor. There is no significant difference between two groups.

**Figure 3:**
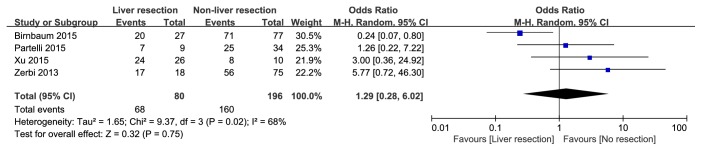
Forest plot for the Grade classification (G1&G2/G3) of liver resection group and nonliver resection group in patients with liver metastases from pancreatic neuroendocrine tumor. There is no significant difference between two groups.

**Figure 4:**
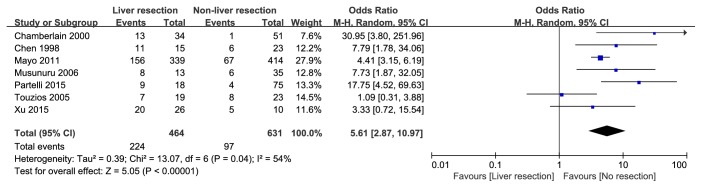
Forest plot for the site of hepatic metastases (unilobar/bilobar) of liver resection group and nonliver resection group in patients with liver metastases from pancreatic neuroendocrine tumor. Liver resection group have more unilobar hepatic metastases.

A total of 23 articles were selected for full-text. Among them, 9 studies were recognized as high quality studies because of NOS score ≥7 and 4 studies lacking some of the key data got 6 points, while the rest, 10 studies, were excluded due to NOS scores< 6. In summary, 13 cohort [13–25] studies were included and the details of the included and excluded [26–35] studies were shown in Table 1 and Supporting Table 1. There were 1524 patients in 13 cohort studies included in this meta-analysis, and 616 patients undergoing hepatic resection were divided into liver resection group while other 908 patients were with nonliver resection therapies as the control group. The details of baseline data from these studies were shown in Table 1. Furthermore, a meta-analysis of the site of hepatic metastases (unilobar/bilobar), the grading system (G1&G2/G3), and the occurrence time of hepatic metastases (synchronous/metachronous) was performed, and there is no significant difference on the occurrence time of hepatic metastases and the grading system between two groups (Figure 2: OR=0.63; 95% CI, 0.24, 1.64;* p*=0.34.  Figure 3: OR=1.29; 95% CI, 0.28, 6.02;* p*=0.75) while the liver resection group have more unilobar hepatic metastases (Figure 4: OR=5.61; 95% CI, 2.87, 10.97;* p*<0.001).

Then a further literature review of 13 included studies was performed. (Table 2) In general, the median postoperative adjuvant therapy rate is of 42.00% (0%-68.00%). For postoperative outcomes, the morbidity was 33.5% (3.28%-44.44%) and the 30-day mortality was 3.32% (0%-5.30%). For long-term outcomes, compared with nonliver resection group whose median OS was 17 months (17-54.8), liver resection group could be prolonged to 84 months (36-123). Overall, almost all studies have an agreement on the idea that hepatic resection could provide a prognosis benefit for PNET patients with liver metastases and according to the data from these studies, there was no concern about the safety of liver resection.

**Figure 5:**
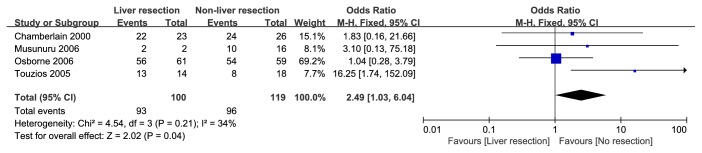
Forest plot for the symptom relief (hormonal symptoms and mechanical symptoms) of liver resection group and nonliver resection group in patients with liver metastases from PNET (functioning or nonfunctioning). Liver resection group have a higher symptom relief rate.

**Figure 6:**
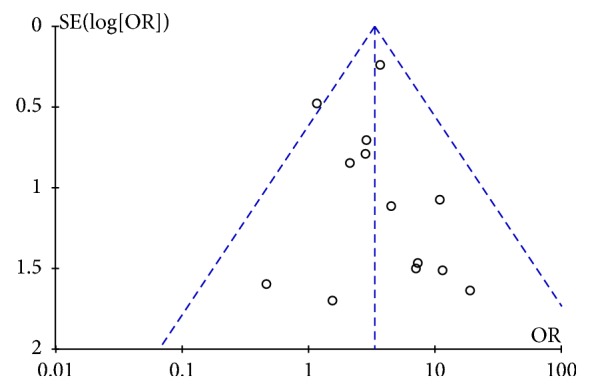
Funnel plot for evaluating publication bias-results from 13 studies.

The median 1-year, 3-year, and 5-year OS rates of all patients in the liver resection group were 92.69%, 76.93%, and 67.54%, respectively, whereas the data of nonliver resection group was 77.31%, 40.94%, and 26.6%. In the retrieved studies, liver resection was not only related to a significant higher 1-year OS rate (OR=3.31; 95% CI, 2.34, 4.67;* p*<0.001), 3-year OS rate (OR=4.29; 95% CI, 2.71, 6.80;* p*<0.001), and 5-year OS rate (OR=5.30; 95% CI, 3.24, 8.67;* p*<0.001) (Table 3) but also created a chance for patients, no matter with functional or nonfunctional PNET, to have a higher symptom relief rate including hormonal symptoms, mechanical symptoms (Figure 5: OR=2.49; 95% CI, 1.03, 6.04;* p*=0.04). A funnel plot of these 13 studies was used to examine publication bias in the meta-analysis (Figure 6). As shown in Figure 6, this plot showed that there was no significant publication bias in this meta-analysis and unpublished data were not evaluated.

## 4. Discussion

Nowadays, there are various treatments for PNET patients with liver metastases including biotherapy, chemotherapy, and intervention. However, no randomized, controlled studies demonstrated that any of them could prolong the OS [7, 36, 37]. Hepatic resection may be the only treatment, even a curative treatment, to prolong the OS. In this meta-analysis, we are mainly intended to compare the prognosis of hepatic resection group with nonliver resection group in PNET patients with liver metastases. According to the result, liver resection is a safe treatment with a low mortality rate (3.32%) and an acceptable morbidity rate (33.5%). The grading system is related to the proliferation capacity of the tumor measured by Ki-67 staining of the PNET specimens. According to previous studies, patients with G1 and G2 PNET have a better survival compared with G3 [38, 39]. In this meta-analysis, there was no difference between two groups about patients with G1, G2, or G3 (*p*=0.75). However, we could not further perform a subgroup analysis about the OS of patients with G1, G2, and G3 because of the lack of data. From 13 included cohort studies, liver resection provided a longer median survival, a higher 1-, 3-, and 5-year survival rate and postoperative symptom relief rate. Notably, 13 studies in our pool showed PNET patients with liver metastases undergoing hepatic resection had a median of 67.54% for 5-year survival rate, which is higher than 60% from previous reports [40].

There was one citation retrieved which suggested that 1-, 3-, and 5-year survival rate in liver resection group were lower than nonliver resection group (95, 93, and 87% versus 87, 84, and 66%, p =0.006) because there were no hepatic metastases in nonliver resection group [23]. The rest 12 studies included had a consensus on a prolonged OS and higher 1-, 3-, and 5-year survival rate in liver resection group and, with extending of follow-up duration, the difference was more obvious between the two groups. Although a conclusion that liver resection could prolong the OS for PNET patients with liver metastases was easily acquired, we could not overlook the fact that liver resection group have more unilobar hepatic metastases which means there were more patients with resectable liver metastases. Touzios et al. even suggested that patients with more than 50% liver involvement may not benefit from a liver resection (5-year survival rate: 67% versus 8%,* p*<0.05) [16]. In another word, liver resection could prolong the OS just for highly selected PNET patients with resectable hepatic metastases.

Due to the decrease of tumor bulk, the symptom relief rate was higher in liver resection group of this meta (*p*=0.04) [14, 16, 17, 19]. Some authors proposed that liver resection should be attempted if at least 90% of visible tumors could be removed, which means cytoreduction, to relieve symptoms for patients with malignant PNET [7, 41, 42]. Three of included studies (Chamberlain et al., Osborne et al., and Partelli et al.) clarified that cytoreduction could even offer a survival benefit [14, 17, 24]. However, in recent years, some authors consider that 90% debulking threshold is completely made up and advocates that 70% debulking threshold might be a better cut-off value. Morgan et al. discovered that there was no difference in OS between patients who had 100%, >90% or > 70% cytoreduction for all 44 PNET patients with liver metastases (*p*=0.75) [43]. Although there were small bowel and PNET for all 108 patients, Maxwell et al. proposed that patients with greater than 70% debulking had significantly improved OS compared with patients with less than 70% (median OS: not reached vs 6.5 years,* p*=0.009) [35]. Some included researches carry out liver resection for PNET patients with extrahepatic metastases but did not conduct indepth research on it [14, 16, 21]. Morgan et al. also did the same, yet all deaths in the series were due to liver failure instead of extra-hepatic disease [43]. Thus, 70% debulking threshold might be able to replace 90% for PNET patients with liver metastases and extra-hepatic metastases should not be an obstacle for surgical therapy [44].

Further, some reviews even suggested that the resection of primary tumors should be attempted even if there were metastases because it might decrease the rate of development of liver metastases and extends survival by preventing the development of progressive disease [7, 45, 46]. In this pooled meta-analysis, 7 studies performed resection of primary tumors in nonliver resection group, but we could not perform a subgroup analysis to support the view because of the lack of data [13, 14, 16, 18, 20, 21, 23]. Although we could not draw a conclusion that palliative resection would provide a better prognosis according to these results, we still should attach importance to the role of palliative resection in advanced PNET patients and launch some prospective researches.

Due to the high recurrence rate even after liver resection [38], postoperative adjuvant therapies, including TACE/HAE, systemic chemotherapy, somatostatin analogues, and PRRT, were recommended for PNET patients with hepatic metastases [47]. In this study, the median postoperative adjuvant therapy rate was 42.0% in liver resection group. Although all postoperative adjuvant therapies could relieve the symptoms, their effects on overall survival have not been proved [7, 17, 48–52]. Recently, everolimus (mammalian target of rapamycin [mTOR] inhibitor) yielded a longer progression-free survival (PFS) and a survival benefit of 6.3 months compared with placebo which brought a hope for advanced PNET patients [37, 53].

Our study has some limitations. There were no randomized controlled trials (RCTs) in retrieved studies and the overall level of clinical evidence was relatively low. The heterogeneity of treatment ranging from the sole resection of the primary tumor to multimodal therapy concepts (somatostatin analogues, chemotherapy, TACE/HAE, and resection of the primary tumor) in nonliver resection, and the heterogeneity of patients' characteristics between two groups might be a source of bias.

## 5. Conclusions

If PNET patients with resectable liver metastases were highly selected, liver surgical resection was an effective and safe treatment to provide a better long-term prognosis including prolonged OS and higher symptom relief. Therefore, there is an urgent need for a further randomized, controlled trial to solve this clinical issue.

## Figures and Tables

**Table 1:** Basic study characteristics of included trials.

**Author**	**Year**	**Country**	**Time Period**	**Patient Number**	**Age**		**Male/Female ratio**	**Grade**	**Hepatic resection (Y/N)**	**Hepatic Metastases**		**NOS score**
					**Liver resection**	**Non-liver resection**		**G1/G2/G3**		**Unilobar/**	**Synchronous/**	
										**Bilobar**	**Metachronous**	
Birnbaum et al	2015	France	1995-2012	118	60 (31-82)	57 (20-83)	62/56	48/43/13	27/91	10/17	27/0	7
Xu et al	2015	China	2008-2013	36	49.9 ± 11.0	56.5 ± 12.5	14/22	6/26/4	26/10	25/11	NR	6
Partelli et al	2015	Italy	2000-2011	93	50 (45-59)	51.5 (43-63)	53/40	21/51/20	18/75	13/80	93/0	7
Zerbi et al	2013	Italy	2004-2007	45	56.9	60.6	26/19	13/19/11	9/36	NR	NR	6
Kleine et al	2011	German	1990-2009	15	55 (20-77)		NR	NR	9/6	NR	15/0	7
Mayo et al	2011	America	1985-2010	753	56 ± 12.6	57 ± 12.8	391/362	NR	339/414	223/530	273/480	8
House et al	2006	America	1988-2003	31	52 (31-71)	41 (31-52)	15/16	NR	26/5	NR	NR	9
Osborne et al	2006	America	2000-2004	120	56 ± 11.6	58 ± 11.1	64/56	NR	61/59	NR	NR	6
Musunuru et al	2006	America	1996-2004	48	56 (27-85)		NR	NR	13/35	14/34	28/20	7
Touzios et al	2005	America	1990-2004	42	58 ± 3	59 ± 3	17/25	NR	19/23	15/27	32/10	6
Solorzano et al	2001	America	1988-1999	100	NR	NR	56/44	NR	20/80	NR	NR	7
Chamberlain et al	2000	America	1992-1998	85	50 (20-79)	54 (23-79)	37/48	NR	34/51	14/71	63/22	7
Chen et al	1998	America	1984-1995	38	54 ± 4	59 ± 3	24/14	NR	15/23	17/21	NR	7

NR indicates no report.

**Table 2:** All included literatures review.

Study ID	Morbidity	30-day mortality	Postoperative adjuvant therapy Rate	Median OS (m)		Median follow-up (m)	Non-liver resection treatments	Conclusion
				Hepatic resection	Non-liver resection			
Birnbaum 2015	44.00%	5.00%	NR	90	NR	NR	Resection of primary tumors	Resection of liver metastases improve survival
Xu 2015	NR	NR	NR	57.2	54.8	32	Somatostatin analogues and chemotherapy	Resection of liver metastases could not prolong OS but could improve PFS
Partelli 2015	44.44%	NR	68.00%	97	36	41	Somatostatin analogues, PRRT, chemotherapy	Resection of liver metastases improve survival
Zerbi 2013	NR	NR	48.68%	NR	20.5	21	Somatostatin analogues, PRRT, ablation, chemotherapy	Resection of liver metastases could be the first-choice treatment for malignent PNET
Kleine 2011	22.22%	NR	NR	NR	37.8	40	Resection of primary tumors	Resection of liver metastases may prolong OS
Mayo 2011	NR	NR	NR	123	33	26	Intra-arterial therapy and resection of primary tumors	Hepatectomy most benefited those patients with low-volume ( <25%) liver metastasis or those with symptomatic high-volume liver metastasis
House 2006	25.00%	0.00%	11.54%	78	17	NR	Somatostatin analogues, chemotherapy, chemoembolization and resection of primary tumors	There is a survival benefit from complete surgical resection of metastatic islet cell tumors originating from the pancreas
Osborne 2006	3.28%	1.64%	65.57%	NR	NR	NR	Somatostatin analogues and chemotherapy, PRRT, embolization	Patients who undergo surgical cytoreduction of symptomatic neuroendocrine hepatic metastases enjoy prolonged survival when compared with their medically treated counterparts
Musunuru 2006	NR	NR	8.00%	NR	NR	20	Systemic hormonal and chemotherapy, ablation, hepatic artery embolization	In patients with liver-only neuroendocrine metastases, surgical therapy is associated with improved survival
Touzios 2005	42.00%	5.30%	36.00%	>96	NR	NR	Somatostatin analogues, chemotherapy, PRRT, ablation and resection of primary tumors	Resection has been shown to be an excellent treatment and accumulating data document improved survival with resection of these tumors
Solorzano2001	NR	NR	60.00%	36	21.6	32	Chemotherapy, hepatic artery embolization	Aggressive management should probably be restricted to younger patients with limited extrahepatic disease
Chamberlain 2000	NR	NR	NR	NR	NR	27	Hepatic artery embolization, Somatostatin analogues and chemotherapy and resection of primary tumors	Hepatic resection has a role in the man-agement of patients with NET metastases and may prolong survival
Chen 1998	NR	NR	0.00%	NR	27	27	Chemoembolization, chemotherapy, PRRT and resection of primary tumors	Hepatic resection for metastatic neuroen-docrine tumors may prolong survival

**Table 3:** Survival outcomes of liver resection group versus nonliver resection group.

**Survival outcomes**	**No. Of studies**	**No. Of event for liver resection**	**No. Of event for non-liver resection**	**OR**	**95**%** CI**	**P Value**	**Heterogeneity ** **P, I**^2^	**Meta-analysis model**
1-year overall survival	13	571/616	702/908	3.31	2.34, 4.67	<0.001	0.55, 0%	Fixed
3-year overall survival	12	467/607	357/872	4.29	2.71, 6.80	<0.001	0.02, 52%	Random
5-year overall survival	12	410/607	232/872	5.30	3.24, 8.67	<0.001	0.02, 53%	Random

## Supplementary Materials

Supplementary Materials Supporting Table 1: Basic study characteristics of excluded cohort studies.
