# Supplementary material for: Resection of Liver Metastases: A Treatment Provides a Long-Term Survival Benefit for Patients with Advanced Pancreatic Neuroendocrine Tumors: A Systematic Review and Meta-Analysis
Source: J Oncol. 2018 Nov 14;2018:6273947. doi: 10.1155/2018/6273947 (PMC6261248; doi:10.1155/2018/6273947)
Supplement: Supplementary Materials — Supporting Table 1: Basic study characteristics of excluded cohort studies. [file 6273947.f1.pdf]

Supporting table 1 Basic study characteristics of excluded cohort studies

First author	Year	Country	Data period	Research Type	Reasons For Excluding	NOS score
Maxwell JE	2016	America	1999-2014	Cohort Study	Lack of intact data	4
Bertani E	2015	Italy	1996-2011	Cohort Study	lack of intact data of pancreatic tumors	3
Du S	2015	China	2004-2014	Cohort Study	Few cases	4
Krausch M	2014	German	2006-2012	Cohort Study	Comparability does not meet current target	3
Poultsides GA	2012	America	1998-2009	Cohort Study	Comparability does not meet current target	3
De Jong MC	2010	America	1970-2008	Cohort Study	Comparability does not meet current target	4
Mayo SC	2010	America	1985-2005	Cohort Study	No selection of controls on the basis of design	4
Elias D	2009	France	2002-2007	Cohort Study	No selection of controls on the basis of design	5
Elias D	2003	France	1985-2000	Cohort Study	No selection of controls on the basis of design	4
Yao KA	2001	America	1992-2000	Cohort Study	Lack of intact data	4
